# Determination of Phenolic Compounds and Antioxidant Activity in Leaves from Wild *Rubus* L. Species

**DOI:** 10.3390/molecules20034951

**Published:** 2015-03-18

**Authors:** Jan Oszmiański, Aneta Wojdyło, Paulina Nowicka, Mirosława Teleszko, Tomasz Cebulak, Mateusz Wolanin

**Affiliations:** 1 Department of Fruit and Vegetable Processing, Wroclaw University of Environmental and Life Science, 37 Chełmońskiego Street, 51-630 Wroclaw, Poland; E-Mails: aneta.wojdylo@up.wroc.pl (A.W.); paulina.nowicka@up.wroc.pl (P.N.); miroslawa.teleszko@up.wroc.pl (M.T.); 2 Faculty of Biology and Agriculture, University of Rzeszow, 4 Zelwerowicza Street, 35-601 Rzeszów, Poland; E-Mails: tomcebulak@gmail.com (T.C.); mwolanin@gmail.com (M.W.)

**Keywords:** LC-PDA/MS, phenolic compounds, ellagitannins, ABTS, FRAP

## Abstract

Twenty-six different wild blackberry leaf samples were harvested from various localities throughout southeastern Poland. Leaf samples were assessed regarding their phenolic compound profiles and contents by LC/MS QTOF, and their antioxidant activity by ABTS and FRAP. Thirty-three phenolic compounds were detected (15 flavonols, 13 hydroxycinnamic acids, three ellagic acid derivatives and two flavones). Ellagic acid derivatives were the predominant compounds in the analyzed leaves, especially sanguiin H-6, ellagitannins, lambertianin C, and casuarinin. The content of phenolic compounds was significantly correlated with the antioxidant activity of the analyzed samples. The highest level of phenolic compounds was measured for *R. perrobustus*, *R. wimmerianus*, *R. pedemontanus* and *R. grabowskii*. The study showed that wild blackberry leaves can be considered a good source of antioxidant compounds. There is clear potential for the utilization of blackberry leaves as a food additive, medicinal source or herbal tea.

## 1. Introduction

Many plants look similar to one another, especially wild plants. In Poland, 63 species of blackberries occur in the wild, but only a few of them have nutritional and healing properties [1,2].

Blackberry leaf has many traditional uses, and it is officially approved in Germany for treating certain health conditions. Blackberry leaves can be made into tea or used as a mouthwash and gargle solution, according to Flora Health [1]. Tannins in blackberry leaf are responsible for some of the beneficial effects, although tannins can cause liver damage if taken in large amounts over long time frames. One should consult a qualified health-care provider before using blackberry leaf supplements. Commission E, the German regulatory agency for herbs, has approved blackberry leaf tea for relieving non-specific acute diarrhea. Tannins in the leaves can alleviate this problem, according to Flora Health. Commission E advises taking 4.5 grams of blackberry leaves daily as a tea or other internal supplement. The University of Maryland Medical Center (UMMC) lists a standard dosage of blackberry leaf tea for relieving diarrhea as 1 heaped teaspoon of dried leaves per cup of hot water, drinking 1/2 cup per hour. The UMMC recommends talking to a doctor before taking blackberry leaf for treating diarrhea, because certain types of diarrhea can be worsened by herbal treatment [3].

Martini *et al.* [4] evaluated the effects of *R. ulmifolius* on *Helicobacter pylori* bacteria, using leaves and isolated polyphenols. *H. pylori* is a common cause of gastrointestinal ulcers and stomach inflammation. It has developed some resistance to antibiotics, and antibiotics for treating *H. pylori* infection are not readily available in developing countries. The leaf extract and all of the polyphenols had antibacterial effects against *H. pylori*. The most important phenolic compounds in blackberry leaves are ellagitannins, which show high antioxidant and free radical scavenging activities. For this reason, their potential effects in preventing oxidative related diseases, such as cardiovascular diseases, have been widely studied. *In vitro* studies show that ellagitannins, at concentrations in the range of 10–100 µM, show some relevant anti-atherogenic, anti-thrombotic, anti-inflammatory and anti-angiogenic effects, supporting the molecular mechanisms for vascular health benefits [5]. There is clear potential for the use of blackberry leaves in the food, cosmetic and pharmaceutical industries.

Among common fruits and vegetables, blackberry is one of the richest in anthocyanins, flavonol glycosides, and other phenolics, which contribute to the high antioxidant capacity of its berries. However, data on the chemical composition of *Rubus* species leaves are scarce. Although the content of leaf phenolics is affected by environmental conditions and the level of maturity at harvest, it is very important to know the chemical composition and the antioxidant capacity of different *Rubus* species in order to selectively use them in the pharmaceutical and alimentary industries.

So far, to our knowledge, there have been no comparative studies on the chemical composition of leaves of a large number of *Rubus* species. Consequently, the purpose of this study was to identify a broad range of phenolic acids and flavonoids and their contents in leaves of 26 species belonging to the *Rubus* genus, and to compare them. This is the first paper about flavonoids and the phenolic acid composition of numerous members of the multispecies *Rubus* genus.

## 2. Results and Discussion

### 2.1. Peak Identification and Assignment

Identification and peak assignment of phenolic compounds in blackberry leaves was based on comparison of their retention times and mass spectral data with those of standards and published data (Table 1). Thirty-three phenolic compounds were detected in wild blackberry leaves. Fifteen of them were flavonols: five kaempferol (MS^2^ ion at *m/z* 285.0187) and 10 quercetin (MS^2^ ion at *m/z* 301.0277) derivatives. By comparing their mass spectral data with those reported previously [6,7,8], these flavonols were tentatively identified as monoglucosides of two quercetin-3-*O*-pentosides (MS ion at *m/z* 433.0777), three quercetin-3-*O*-hexosides (MS ion at *m/z* 463.0843) and quercetin-3-*O*-glucuronide (MS ion at *m/z* 447.0968). Other quercetin derivatives such as 3-methoxyhexoside (MS ion at *m/z* 493.1001), -3-*O*-rutinoside (MS ion at *m/z* 609.1080) and -3-(6''-(3-hydroxy-3-methylglutaroyl)-galactoside (MS ion at *m/z* 607.1293) and quercetin-3-*O*-6-acetylglucoside (MS ion at *m/z* 505.0980) were identified according to previously published data [6,7,8,9,10]. Five kaempferol derivatives were identified:-3-*O*-glucoside-rhamnoside-7-*O*-rhamnoside with *m/z* 739.1930 and MS/MS fragments at 593.1559 obtained after the loss of 146 amu (rhamnose moiety) and MS/MS fragments at 285.0187 after the loss of 308 amu (rhamnoglucoside moiety), -3-*O*-rutinoside (MS ion at *m/z* 593.1559), -3-*O*-glucuronide (MS ion at *m/z* 461.0710), -3-*O*-6-acetylglucoside (MS ion at *m/z* 489.1042) and a non-identified kaempferol derivative (MS ion at *m/z* 475.0271 with MS fragmentation ion at *m/z* 447.0968 and 285.0187). Cho *et al.* [9,11] reported the presence of these kaempferol derivatives in blackberry samples.

Two flavones were detected in wild blackberry fruit extracts: luteolin-3-*O*-glucuronide (MS ion at *m/z* 461.0710 with MS fragmentation ion at *m/z* 285.0187) and apigenin-3-*O*-glucuronide (MS ion at *m/z* 445.0710 with MS fragmentation ion at *m/z* 269.0450). These compounds had maximum absorption at shorter wavelengths (340 nm and 338 nm) than flavonols, which indicated their presence in the analyzed samples.

Nine phenolic acid derivatives were detected in the blackberry leaf extracts. Among them were: neochlorogenic, chlorogenic acid and *p*-coumaric acid, identified by comparison with standard compounds. Two caffeoyl hexosides were found with *m/z* 341.0849 and an MS/MS fragment at 179.0349, obtained after the loss of 162 amu (hexose moiety) [10,12]. The caffeoyl dihexose and caffeic acid derivatives were identified with *m/z* 503.1190 and *m/z* 459.094, respectively. These two compounds had a spectrum characteristic for caffeic acid derivatives, with ë_max_ at 324 nm. The *p*-coumaroylquinic acid was identified with *m/z* 337.0937 and fragmentation *m/z* 191.0553 (as quinic acid) and *m/z* 163.0380 (as *p*-coumaric acid).

Some ellagitannin and ellagic acid derivatives were identified in *Rubus* leaves. Ellagic acid (MS ion at *m/z* 300.9999) and ellagic acid pentoside (MS ion at *m/z* 433.0777), rhamnoside (MS ion at *m/z* 447.0527) and methyl ellagic acid pentose (MS ion at *m/z* 477.1082) were identified in blackberry extracts based on mass spectral data and comparison of their retention times with those of standards and published data (Table 1) [10,12,13]. Three ellagitannins—sanguiin H-6 (MS ion at *m/z* 1869.0851), lambertianin C (MS ion at *m/z* 1401.3730) and ellagitannins hexoside (casuarinin) (MS ion at *m/z* 935.0760)—were identified in wild blackberry leaf based on maximum absorption at 240 nm, mass fragmentation spectral data *m/z* 633.0750 (galloyl-hexahydroxydiphenoyl–glucose; galloyl-HHDP-glucose) and *m/z* 300.9999 (ellagic acid), and published data [14,15]. Sanguiin H-6 (comprising four hexahydroxydiphenoyl, two galloyl and two glucosyl units) is the major ellagitannin in berries and their products [16]. Lambertianin C consists of six hexahydroxydiphenoyl, three galloyl and three glucosyl moieties [17]. Lambertianin C is relatively abundant in *Rubus* fruit [18].

**Table 1:** The characterization of phenolic compounds in blackberry leaves, using their spectral characteristics in UPLC-PDA (retention time, λ_max_) and negative ions in LC-QTof/MS.

Compounds	R_t_ (min)	λ_max_ (nm)	[MS]^−^	[MS-MS]^−^
*p*-Coumaric acid derivative	1.35	314	787.9050	420.9105/347.9189/163.0380
Neochlorogenic acid	2.27	323	353.0866	235.9249/191.0553/146.9378
Chlorogenic acid	2.35	323	353.0866	235.9249/191.0553/146.9378
Caffeoyl hexoside	2.99	320	341.0849	179.0349/135.0464
*p*-Coumaroylquinic acid	3.14	314	337.0937	191.0553/163.0380
Caffeoyl hexoside	3.45	320	341.0849	179.0349/135.0464
*p*-Coumaric acid	4.22	312	163.0380	
Sanguiin H-6	4.79	245	1869.0851	935.0760/633.075/300.9999
Ellagitannins Lambertianin C	5.03	244	1401.3730	633.075/300.9999
Ellagitannins hexoside (casuarinin)	5.53	244	935.0760	633.075/300.9999
Ellagic acid pentoside	6.28	360	433.0777	300.9999
Quercetin-3-methoxyhexoside	6.38	360	493.1001	463.3010
Ellagic acid	6.51	364	300.9999	
Ellagic acid rhamnoside	6.64	360	447.0527	300.9999
Kaempferol-3-*O*-glucoside-rhamnoside-7-*O*-rhamnoside	6.73	346	739.1930	593.1559/285.0187
Quercetin-3-*O*-rutinoside	6.90	352	609.1080	463.0397/301.0277/151.0034
Quercetin-3-*O*-galactoside	7.04	353	463.0843	301.0277/151.0034
Quercetin-3-*O*-glucuronide	7.14	351	477.0670	301.0277/151.0034
Quercetin-3-*O*-glucoside	7.20	352	463.0843	301.0277/151.0034
Kaempferol derivative	7.27	345	475.0753	447.0968/285.0187
Quercetin-3-*O*-hexoside	7.32	352	463.0843	301.0277/151.0034
Luteolin-3-*O*-glucoronide	7.49	340	461.0710	285.0187
Quercetin-3-*O*-pentoside	7.88	352	433.0777	301.0277/151.0034
Quercetin-3-[6''-(3-hydroxy-3-methylglutaroyl)-galactoside	7.94	345	607.1293	463.0843/301.0277/151.0034
Quercetin-3-*O*-pentoside	8.12	352	433.0777	301.0277/151.0034
Kaempferol-3-*O*-rutinoside	8.27	350	593.1559	447.0968/285.0187
Kaempferol-3-*O*-glucuronide	8.43	346	461.0710	285.0187
Methyl ellagic acid pentose	8.6	360	477.1082	314.0421/300.9996
Quercetin-3-*O*-6-acetylglucoside	8.76	350	505.0980	447.0397/301.0277/151.0034
Apigenin-3-*O*-glucoronide	8.90	338	445.0710	269.0450
Caffeoyldihexoside	9.20	324	503.1190	341.0773/179.0321
Caffeic acid derivative	9.66	324	459.094	179.0321/161.0241
Kaempferol-3-*O*-6-acetylglucoside	10.19	345	489.1042	284.0313

Rt–retention time.

### 2.2. Phenolic Compounds from Wild Blackberry Leaves

**Figure 1:**
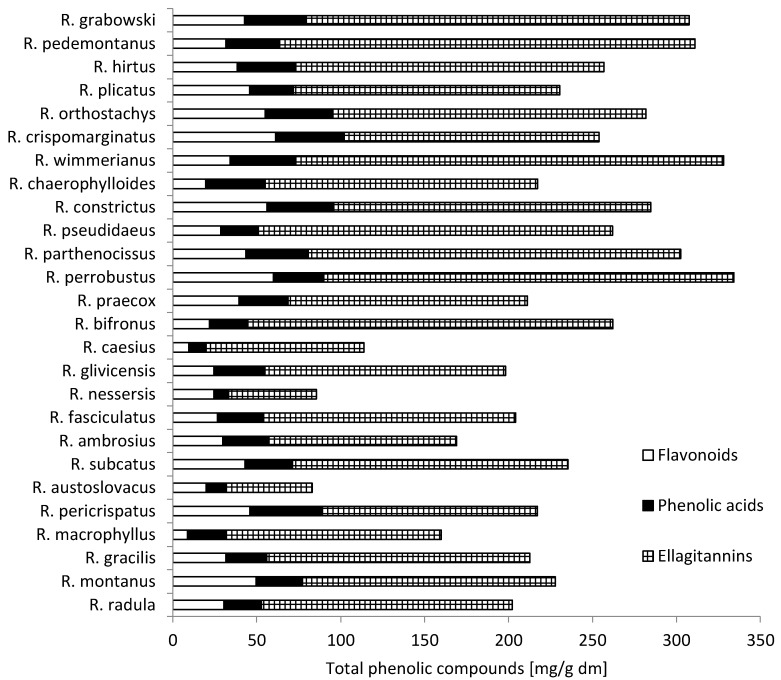
Content of total phenolic compounds in leaves [mg/g dm] from wild *Rubus* L. species.

Analysis of the extracted phenolic compounds of 26 samples is presented in Figure 1. The total content of flavonoid derivatives, phenolic acids and ellagitannins was calculated as the sum of compounds resulting from UPLC-PDA analysis. The total content of phenolic compounds extracted from leaves of wild blackberry was highly diverse and ranged from 83.02 mg/g dry matter (dm) for *R. austroslovacus* to 334.24 mg/g dm for *R. perrobustus*. The *R. pomobustus*, *R.* *wimmerianus*, *R. grabowskii*, and *R. pedemontaneus* samples had the highest content of phenolics. The *R.* *austroslovacus*, *R. nessensis*, and *R. caesius* samples had the lowest content.

Among particular groups of phenolic compounds, the largest was composed of ellagitannins—from 51.59 mg/g dm for *R. austroslovacus* to 255.01 mg/g dm for *R. wimmerianus*. Blackberries, especially their leaves, are known for their high content of ellagitannins, which determine their value in the prevention of diseases [5]. The average content in all analyzed samples was 165.84 mg/g dm.

The second important group of bioactive compounds contained in the leaves of wild blackberries was composed of flavonoid derivatives of quercetin, kaempferol, luteolin and apigenin. The content of these compounds ranged from 8.68 mg/g dm in the leaves of *R. macrophyllus* to 61.27 mg/ g dm in leaves of *R. crispomarginatus*. Their average content in all analyzed samples of leaves was 35.17 mg/g dm. Gudej and Tomczyk [19] found the highest flavonoid aglycone content after hydrolysis in the wild leaves of *R. nessensis* (1.06% dm), and the lowest in the wild leaves of *R. fruticosus* (0.34% dm), respectively. These compounds also play an important role as substances with a high antioxidant activity and in the prevention of many diseases [20]. Significantly greater differentiation between wild species of blackberry leaves occurred in the presence of flavonoids. Only seven compounds, from 17 flavonoids, were identified in all species. A rarely occurring flavonol amongst the examined species was kaempferol-3-*O*-glucoside-rhamnoside-7-*O*-rhamnoside. It was observed in only nine out of 26 species, while kaempferol derivative was observed only in two species, quercetin-3-[6''-(3-hydroxy-3-methylglutaroyl)-galactoside] in 13 species and quercetin-3-*O*-pentoside in 14 species.

The most abundant compounds of flavonoids were kaempferol-3-*O*-glucuronide (average of all species 9.23 mg/g dm), and quercetin-3-*O*-glucuronide (average of all species 7.00 mg/g dm). In nine species of wild blackberry leaves, quercetin-3-*O*-glucuronide was not detected, and the largest amount of this compound was in *R. crispomarginatus* (28.08 mg/g dm).

The next group of polyphenols in blackberries is composed of phenolic acids, derivatives of caffeic acids, *p*-coumaric acids and ellagic acids. The amount of these compounds found ranges from 8.62 mg/g dm in leaves of *R. nessensis* to 43.14 mg/g dm in leaves of *R. pericrispatus*. The average from all samples of blackberry leaves was 28.74 mg/g dm. Among these compounds, the content of ellagic acid and its derivatives is especially valuable, because these compounds are assigned anti-tumor activity [21].

Leaves from wild *Rubus* L. species significantly differed in qualitative and quantitative composition of individual compounds from the group of phenolic acids and ellagitannins (Table 2) and flavonoids (Table 3). In the species poorest in polyphenolic compounds, *R. austroslovacus*, one of the ellagitannins, sanguiin H-6, was not detected. Moreover, two flavonoids, quercetin-3-*O*-glucuronide and kaempferol derivative, were not detected either. In the leaves of *R. perrobustus*, the species most rich in polyphenol compounds, the presence of five compounds present in other species was not detected: ellagic acid rhamnoside, kaempferol-3-*O*-glucoside-rhamnoside-7-*O*-rhamnoside, quercetin-3-*O*-glucoside, kaempferol derivative, quercetin-3-[6''-(3-hydroxy-3-methylglutaroyl)-galactoside and quercetin-3-*O*-pentoside.

Among derivatives of caffeic acid, in the majority of species, the content of neochlorogenic acid was higher than that of chlorogenic acid. In *R. parthenocissus* the value of neochlorogenic acid was 22.07 mg/g dm, and that of chlorogenic acid only 0.41 mg/g dm. Among derivatives of ellagic acid, ellagic acid rhamnoside was found in the smallest amounts in the leaves of analyzed *R. praecox*, *i.e.*, 0.01–0.21 mg/g dm, while in *R. bifronus*, this compound was not detected. Similarly, small amounts of methyl ellagic acids pentose (from 0.03–0.77 mg/g dm) were found, and in five species these compounds were not detected.

**Table 2:** Individual quantities (mean †, mg/g dm) of each phenolic acid and ellagitannin in different leaves of wild *Rubus* L. species.

Blackberry Species	Phenolic Acid													Ellagitanins		
	pCA der	NChA	ChA	C-hex	*p*-CqA	Chex	p-CA	EAp	EA	EArha	mEApen	Cdihex	CAd	SH6	ELC	Ehex
*R. radula*	4.07 †	7.64	0.39	0.82	2.72	1.77	1.28	0.83	2.33	0.06	0.12	0.18	0.27	16.66	71.08	61.56
*R. montanus*	4.65	8.68	0.44	3.27	3.24	3.55	1.71	0.00	1.56	0.09	0.11	0.06	0.23	16.95	66.43	67.20
*R. gracilis*	3.52	8.14	1.86	2.32	1.85	3.84	0.72	0.24	1.43	0.04	0.12	0.30	0.11	18.07	71.06	67.43
*R. macrophyllus*	5.11	11.07	0.55	0.23	1.91	1.56	0.68	0.34	1.12	0.04	0.05	0.18	0.07	14.48	46.92	66.84
*R. pericrispatus*	3.36	17.50	0.80	4.50	6.01	5.47	3.05	0.20	1.18	0.03	0.15	0.30	0.60	14.49	55.51	58.06
*R. austoslovacus*	2.57	3.56	0.19	0.72	1.37	1.02	0.40	0.40	1.24	0.02	0.06	0.01	0.09	nd	16.75	34.84
*R. subcatus*	5.71	6.12	0.88	4.40	1.68	4.47	1.79	0.25	2.02	nd	0.55	0.07	0.15	59.79	44.99	59.36
*R. ambrosius*	4.43	9.06	0.68	4.69	0.92	4.81	0.75	0.08	1.61	nd	0.30	0.11	0.03	21.11	39.37	51.24
*R. fasciculatus*	5.73	0.89	4.40	1.96	5.26	3.13	1.42	1.31	1.67	0.03	0.13	1.09	0.28	23.24	62.66	64.38
*R. nessersis*	2.29	0.65	1.02	0.26	0.40	0.64	0.52	0.62	2.10	0.01	0.07	0.02	0.03	12.22	5.69	34.47
*R. glivicensis*	4.33	7.30	5.26	1.48	3.58	4.14	2.60	0.33	1.16	0.02	0.10	0.19	0.09	48.46	36.29	58.50
*R. caesius*	5.06	0.74	0.44	0.31	0.93	0.26	0.11	0.85	1.25	0.06	0.04	0.09	0.04	5.79	36.26	51.99
*R. bifronus*	4.31	12.44	0.23	0.32	1.34	1.98	0.72	0.09	1.52	nd	0.03	0.06	0.01	39.48	63.73	114.07
*R. praecox*	4.64	1.45	0.26	7.39	2.34	8.90	0.78	0.33	1.76	nd	0.16	0.66	0.49	18.49	52.36	71.61
*R. perrobustus*	3.16	4.39	1.06	6.82	3.85	6.65	1.61	0.18	1.59	nd	0.32	0.08	0.10	53.02	123.41	67.96
*R. parthenocissus*	3.79	22.07	0.41	2.98	1.23	4.01	0.40	0.26	1.63	nd	0.12	0.13	0.04	11.41	95.06	115.44
*R. pseudidaeus*	3.12	1.33	2.38	0.75	3.59	5.40	0.36	3.30	1.61	nd	0.21	0.48	0.04	15.07	78.00	117.86
*R. constrictus*	5.91	7.69	1.92	6.57	3.02	8.69	1.12	0.27	2.94	nd	0.77	0.40	0.38	24.38	61.83	102.64
*R. chaerophylloides*	3.86	3.93	6.34	3.18	4.54	7.97	1.97	0.22	1.80	nd	0.12	1.44	0.13	13.96	44.72	103.46
*R. wimmerianus*	3.84	15.80	2.67	3.31	4.64	4.38	1.36	0.27	2.42	nd	0.07	0.30	0.11	64.44	76.12	114.46
*R. crispomarginatus*	2.73	6.82	0.38	7.54	3.10	12.11	1.71	0.54	2.85	nd	0.14	1.32	1.45	7.38	60.16	84.43
*R. orthostachys*	4.47	3.96	2.80	6.03	3.92	7.21	1.51	4.07	2.40	nd	nd	3.13	0.43	45.60	57.01	84.12
*R. plicatus*	4.82	5.68	1.52	3.62	1.15	5.83	0.54	0.35	1.74	nd	nd	0.41	0.16	58.48	42.17	58.14
*R. hirtus*	3.35	5.48	1.82	0.93	4.93	11.87	3.13	0.63	1.53	nd	nd	0.37	0.65	73.92	34.67	75.00
*R. pedemontanus*	3.17	5.00	5.27	1.07	8.28	2.97	2.68	0.44	1.56	0.05	nd	0.53	0.42	63.51	71.67	112.73
*R. grabowski*	4.92	2.12	2.49	6.49	6.50	7.69	1.91	0.37	2.09	0.21	nd	0.72	0.54	49.77	64.84	114.00
ANOVA *P* value	***	*	**	*	**	*	***	**	***	***	***	**	**	*	*	*

*p*CAder—*p*-coumaric acid derivative; NChA—neochlorogenic acid; ChA—chlorogenic acid; C-hex—Caffeoyl hexoside; *p*-CqA—*p*-Coumaroylquinic acid; C-hex—Caffeoyl hexoside; *p*-CA—*p*-Coumaric acid; Cdih—Caffeoyldihexoside; Cad—Caffeic acid derivative; EApen—Ellagic acid pentoside; EA—Ellagic acid; Ear—Ellagic acid rhamnoside; mEApen—Methyl ellagic acid pentose; SH6—Sanguiin H-6; ELC—Ellagitannins Lambertianin C; Ehex—Ellagitannins hexoside (casuarinin); † mean value n = 3; nd—not detected; Significant at *p* < 0.05 (*), *p* < 0.001 (**), and *p* < 0.0001 (***).

**Table 3:** Individual quantities (mean †, mg/g dm) of each flavonoids in different leaves of wild *Rubus* L. species.

Blackberry Species	Flavonoids																
	Q-m-hex	K-glu-rha-rha	Q-rut	Q-gal	Q-gluc	Q-glu	Kd	Q-hex	L-gluc	Q-pen	Q-m-gal	Q-pen	K-rut	K-gluc	Q-a-glu	A-gluc	K-a-glu
*R. radula*	0.79	0.21	0.14	0.19	nd	6.43	nd	0.70	1.63	0.51	0.22	0.17	0.48	11.56	0.59	6.60	0.21
*R. montanus*	nd	nd	0.09	0.91	nd	23.87	nd	0.85	6.03	3.54	0.39	0.11	0.21	11.07	1.04	1.16	0.36
*R. gracilis*	0.22	nd	1.16	1.36	nd	7.36	nd	1.54	2.35	0.99	1.24	0.44	1.04	7.03	0.89	5.34	0.61
*R. macrophyllus*	0.27	0.06	0.08	0.04	0.06	0.73	nd	0.04	0.40	0.09	0.03	0.07	0.33	1.25	0.03	5.17	0.03
*R. pericrispatus*	0.16	nd	0.44	0.84	nd	18.91	nd	2.50	5.56	2.68	0.83	0.03	0.57	11.07	0.90	1.46	0.04
*R. austoslovacus*	0.14	0.04	0.22	0.24	nd	2.67	nd	0.12	0.60	0.49	0.08	0.02	0.25	6.03	0.27	8.56	0.04
*R. subcatus*	0.19	0.03	0.25	0.73	nd	10.81	nd	2.39	7.31	2.29	0.92	0.03	0.99	7.33	3.55	3.13	3.19
*R. ambrosius*	nd	nd	0.32	0.49	0.41	7.98	nd	0.28	6.88	1.60	0.37	0.23	0.20	5.08	0.93	4.81	0.19
*R. fasciculatus*	0.30	0.08	0.09	0.12	nd	5.09	3.10	2.56	1.58	0.35	0.12	0.03	0.35	9.30	0.31	3.04	0.11
*R. nessersis*	nd	0.01	0.16	0.07	nd	0.71	nd	0.20	0.60	0.18	0.21	0.34	0.58	1.93	0.05	19.03	0.43
*R. glivicensis*	0.17	0.15	0.07	0.30	nd	5.56	nd	0.33	1.17	0.54	0.18	0.12	0.11	10.81	0.52	4.30	0.08
*R. caesius*	0.35	0.06	0.07	0.02	0.04	1.12	0.79	0.63	0.56	0.16	0.02	0.02	0.13	1.46	0.09	4.02	nd
*R. bifronus*	0.27	0.07	0.20	0.11	0.13	4.96	nd	0.12	1.73	0.48	0.08	0.05	0.49	8.12	0.40	4.56	nd
*R. praecox*	0.22	nd	0.38	0.64	22.26	nd	nd	1.03	0.47	1.91	nd	0.09	0.58	9.29	0.54	1.96	0.16
*R. perrobustus*	0.22	nd	0.25	0.55	17.63	nd	nd	9.89	5.78	2.61	nd	nd	2.67	11.46	4.27	1.25	3.45
*R. parthenocisus*	0.12	nd	0.31	0.58	12.94	nd	nd	1.13	2.84	2.02	nd	nd	0.55	17.88	1.51	3.51	0.16
*R. pseudidaeus*	nd	nd	0.05	0.18	8.69	1.96	nd	3.68	0.23	1.27	nd	nd	0.26	9.43	1.26	1.49	nd
*R. constrictus*	nd	nd	0.55	0.59	15.83	4.58	nd	nd	9.35	2.72	nd	nd	3.85	10.51	3.13	2.36	2.64
*R. chaerophylloides*	0.19	nd	0.30	0.28	7.57	1.67	nd	nd	0.35	0.20	nd	nd	0.52	6.39	0.14	1.74	0.20
*R. wimmerianus*	0.40	nd	0.06	0.13	3.52	nd	nd	2.07	0.90	0.34	nd	nd	2.13	10.68	1.55	7.60	4.66
*R. crispomarginatus*	0.28	nd	0.59	1.84	28.08	nd	nd	3.51	4.66	5.11	nd	nd	0.50	13.67	1.10	1.92	nd
*R. orthostachys*	nd	nd	nd	0.65	24.59	4.64	nd	nd	3.63	4.90	nd	nd	0.33	12.60	0.76	3.01	nd
*R. plicatus*	0.20	nd	2.13	2.99	11.20	3.84	nd	nd	9.84	2.66	nd	nd	1.57	4.90	3.55	1.39	1.63
*R. hirtus*	0.22	nd	0.24	0.05	8.09	4.30	nd	nd	0.26	0.12	nd	nd	0.46	16.51	0.12	8.13	nd
*R. pedemontanus*	0.42	nd	0.05	0.10	3.52	0.87	nd	nd	0.85	0.14	nd	nd	0.17	11.66	0.30	13.52	nd
*R. grabowski*	0.11	nd	0.46	0.72	19.10	nd	nd	1.15	3.49	2.65	nd	nd	0.35	11.40	0.82	2.55	nd
ANOVA *P* value	**	*	*	*	*	*	**	**	**	*	*	*	*	***	***	***	**

Q-m-hex—Quercetin-3-methoxyhexoside; K-glu-rha-rha—Kaempferol-3-*O*-glucoside-rhamnoside-7-*O*-rhamnoside; Q-rut—Quercetin-3-*O*-rutinoside; Q-gal—Quercetin-3-*O*-galactoside; Q-gluc—Quercetin-3-*O*-glucuronide; K-a-glu—Kaempferol-3-*O*-6-acetylglucoside; Q-glu—Quercetin-3-*O*-glucoside; Kd—Kaempferol derivative; Q-hex—Quercetin-3-*O*-hexoside; L-gluc—Luteolino-3-*O*-glucoronide; Q-pen—Quercetin-3-*O*-pentoside; Q-m-gal—Quercetin-3-[6''-(3-hydroxy-3-methylglutaroyl)]-galactoside; K-rut—Kaempferol-3-*O*-rutinoside; K-gluc—Kaempferol-3-*O*-glucuronide; Q-a-glu—Quercetin-3-*O*-6-acetylglucoside; A-gluc—Apigenin-3-*O*-glucuronide; † mean value n = 3; nd—not detected; Significant at *p* < 0.05 (*), *p* < 0.001 (**), and *p* < 0.0001 (***).

### 2.3. Antioxidant Activity

**Figure 2:**
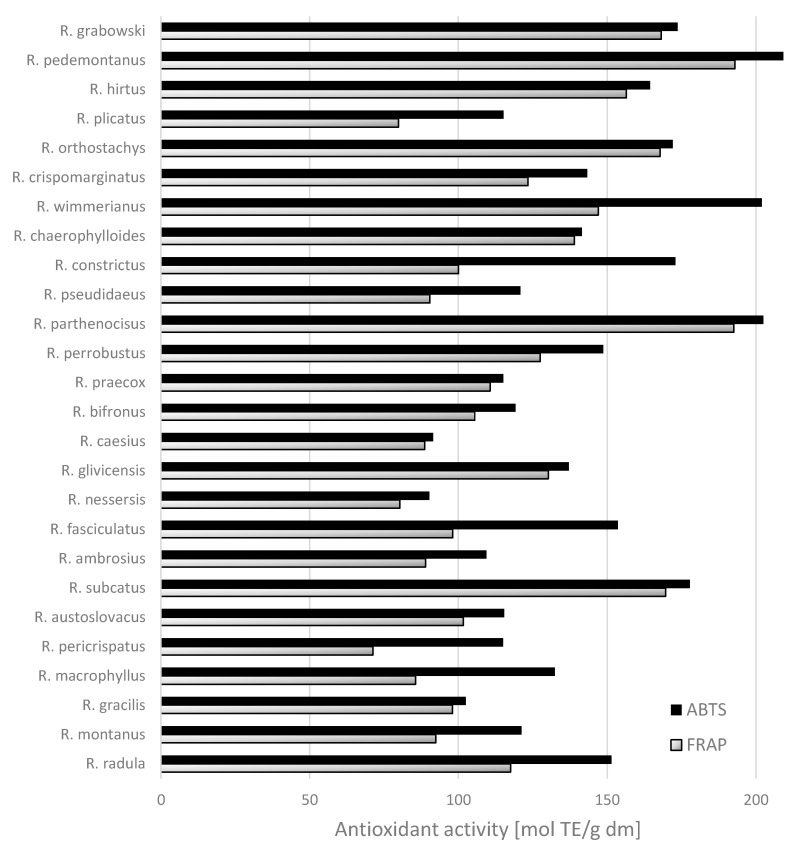
Antioxidant activity evaluated as ABTS^°+^ and FRAP in leaves [mmol TE/g] from wild *Rubus* L. species.

Antioxidant activity of wild blackberry leaf extracts, as measured by FRAP and ABTS^°+^ methods, is presented in Figure 2. The tested extracts from the leaves of wild blackberry species were characterized by diverse antioxidant activity. The lowest FRAP activity was observed for samples of extracts from the leaves of *R. pericrispatus* < *R. plicatus* < *R. nessersis < R. macrophyllus* and the lowest ABTS^°+^ activity for *R. nessersis* and *R. caesius* (Figure 2). The last two species are among those having the lowest contents of phenolic compounds too (Figure 1). In contrast, the highest FRAP abilities were exhibited by the species *R. pedemontanus* (192.91 mmol TE/g dm) and *R. parthenocissus* (192.53 mmol TE/g dm). Both are among the species with a very high content of phenolic compounds (Figure 1). The sample from the leaves of *R.* *pedemontanus* species also showed the highest ability of ABTS radical scavenging (212.69 mmol TE/g dm). Mullen *et al.* [17] reported that sanguiin H-6 was a major contributor to the antioxidant capacity of raspberries fruits, together with Vitamin C and the anthocyanins. The correlation observed between antiradical activity measurements and ellagitannins indicated that phenolics of high molecular weight were major contributors to antioxidant capacity.

Significant positive correlations were found between the results of antioxidant assays (FRAP and ABTS^°+^) and ellagitannins (Pearson correlation = 0.614 and 0.725, respectively) and with total phenolic compounds (Person correlation = 0.608 and 0.737, respectively). The correlation coefficients between the other phenolic compounds and antioxidant activity were weak. Our results indicated that these compounds contributed markedly to the total antioxidant capacity of the leaf samples studied. This can be attributed to the structures of ellagitannins characterized by the presence of several *hydroxy* functions in *ortho* position, which exhibit a greater ability to donate a hydrogen atom and to support the unpaired electron as compared to phenolics of low molecular weight [22]. The leaves of blackberries are rich in phenolics, which have antioxidant and anticancer properties [23,24]. Wang *et al.* [6] found that the leaves from blackberry, raspberry, and strawberry plants had high antioxidant capacities and total phenolics content compared to their fruit tissues; therefore, they reported that *Rubus* leaves have great capacities as free radical scavengers and peroxide decomposers. The obtained results enabled the estimation of the leaf content of phenolics and antioxidant activity across a wide range of species of wild blackberry. These may be used in the preparation of infusions in preventative medicine [23]. Oliveira *et al.* [25] reported that *Cydonia oblonga* Miller leaves had a very high total phenolics content, varying from 4.9–16.5 g/kg dm, and were characterized by higher relative contents of kaempferol derivatives than fruits (pulps, peels, and seeds). Tavares *et al.* [26] demonstrated that the ingestion of wild blackberry species attenuated degenerative processes in the brain, with these benefits ascribed to the phenolic components.

### 2.4. Cluster Analysis

Cluster analysis is an unsupervised data analysis method, meaning that prior knowledge of the sample is not required. HCA enables interpretation of the results in a fairly intuitive, graphic way.

**Figure 3:**
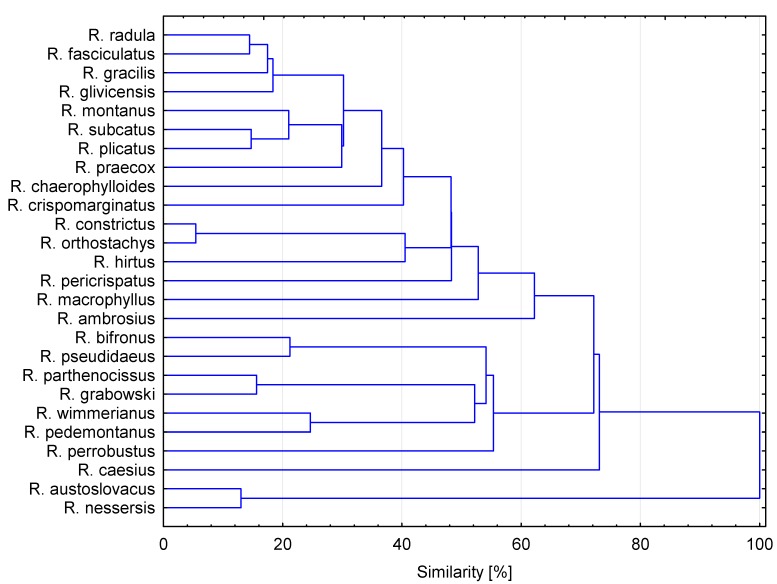
Hierarchical cluster analysis of wild *Rubus* species based on group average cluster analysis of the phenolic compounds profile.

Cluster analysis of the different blackberry leaf samples, according to their phenolic compounds (33 variables), was used as an additional exploratory tool to assess heterogeneity among different quality parameters of *Rubus* leaves. Generally, HCA showed 11 clear similarity clusters (Figure 3). The highest similarity of blackberry species was obtained between *R. nessensis* and *R. austroslovacus*. The lowest similarity (below 30%) was obtained between *R. radula*, *R. fasciculatus*, *R. gracilis*, *R. glivicensis*, *R. montanus*, *R. subcatus*, *R. crispomarginatus* and *R. praecox*. The rest of the analyzed *Rubus* leaves showed similarities between 36% and 74%.

## 3. Experimental Section

### 3.1. Reagents and Standards

Trolox (6-hydroxy-2,5,7,8-tetramethylchroman-2-carboxylic acid), acetic acid, TPTZ (2,4,6-tripyridyl-1,3,5-triazine), 2,2'-azinobis-(3-ethylbenzthiazoline-6-sulfonic acid) (ABTS), FeCl_3_, phloroglucinol, ascorbic acid, acetonitrile, formic acid, and methanol were purchased from Sigma-Aldrich (Steinheim, Germany). Quercetin-3-*O*-glucoside, quercetin-3-*O*-galactoside, kaempferol-3-*O*-glucoside, luteolin-3-*O*-glucoside, apigenin-3-*O*-glucoside, chlorogenic acid, neochlorogenic, ellagic acid, *p*-coumaric acid, ellagic acid were purchased from Extrasynthese (Lyon, France).

### 3.2. Plant Material

Twenty six different wild blackberry leaf samples were collected in September and October 2013 from various localities throughout southeastern Poland (Table 4). Leaves were directly frozen in liquid nitrogen and freeze-dried (24 h; Alpha 1–4 LSC, Christ, Germany). The homogeneous powders were obtained by crushing the dried tissues using a closed laboratory mill to avoid hydration. Powders were kept in a refrigerator (−80 °C) until extract preparation, no longer than 7 days.

**Table 4:** Information about sample area of wild blackberry leaf harvesting.

Blackberry Species	Origin	Geographical Location
*R. radula*	Albigowa Honie	N 50°0’19.28” E 22°10’22.06”
*R. montanus*	Berendowice	N 49°40’14.85” E 22°43’39.58”
*R. gracilis*	Las Niechciałka	N 50°5’45.38” E 22°35’45.06”
*R. macrophyllus*	Las Niechciałka	N 50°5’45.38” E 22°35’45.06”
*R. pericrispatus*	Kopystno	N 49°41’8.38” E 22°38’32.49”
*R. austroslovacus*	Długie k/Przemyśla	N 49°45’49.61” E 22°42’4.59”
*R. sulcatus*	Łazy k/Birczy	N 49°42’49.56” E 22°32’3.14”
*R. ambrosius*	Zmysłówka	N 50°9’58.91” E 22°22’43.39”
*R. fasciculatus*	Łazy k/Birczy	N 49°42’49.56” E 22°32’3.14”
*R. nessensis*	Las Niechciałka	N 50°5’45.38” E 22°35’45.06”
*R. glivicensis*	Zmysłówka	N 50°9’58.91” E 22°22’43.39”
*R. caesius*	Długie k/Przemyśla	N 49°45’49.61” E 22°42’4.59”
*R. bifrons*	Berendowice	N 49°40’26.44” E 22°43’6.76”
*R. praecox*	Ławy k/Birczy	N 49°42’49.56” E 22°32’3.14”
*R. perrobustus*	Łazy k/Birczy	N 49°42’49.56” E 22°32’3.14”
*R. parthenocissus*	Berendowice	N 49°40’26.44” E 22°43’6.76”
*R. pseudidaeus*	Białobrzeszki	N 50°7’18.26” E 22°31’29.98”
*R. constrictus*	Berendowice	N 49°40’14.85” E 22°43’39.58”
*R. chaerophylloides*	Gruszowa	N 49°40’27.7” E 22°41’36.99”
*R. wimmerianus*	Zmysłówka	N 50°9’58.91” E 22°22’43.39”
*R. crispomarginatus*	Łazy k/Birczy	N 49°42’49.56” E 22°32’3.14”
*R. orthostachys*	Berendowice	N 49°40’14.85” E 22°43’39.58”
*R. plicatus*	Łazy k/Birczy	N 49°42’49.56” E 22°32’3.14”
*R. hirtus*	Kolbuszowa	N 50°15’12,63” E 21°47’46,61”
*R. pedemontanus*	Zmysłówka	N 50°9’58.91” E 22°22’43.39”
*R. grabowskii*	Zmysłówka	N 50°9’58.91” E 22°22’43.39”

### 3.3. Extraction Procedure by Pressurized Liquid Extraction (PLE)

The Speed Extractor E-916 (BUCHI Labortechnik AG Switzerland) was used for pressurized solvent extraction. Blackberry leaf powders (0.3 g) were mixed with 1 g of diatomaceous earth and placed into 10 mL extraction cells containing a cellulose paper filter at the bottom of each cell. The cells containing the samples were placed into the accelerated solvent system (ASE system), pre-filled with extraction solvent, pressurized and then heated. The extraction conditions and process were as follows: firstly, a static time of 5 min, followed by a flush elution with a 60% volume, followed by a nitrogen purge of 60 s, then the samples were extracted twice. The extraction was conducted under the following conditions: solvent: 50% methanol acidified with 1% acetic acid; extraction volume: 25 mL; temperature: 50 °C; pressure: 100 bar. As a result, six samples were processed in one run in exactly the same conditions. Extraction was repeated five times. The diluted extracts were filtered through a hydrophilic PTFE 0.20 µm membrane (Millex Samplicity Filter, Merck, Damrstadt, Germany) and then subjected to UPLC-PDA-MS analysis.

### 3.4. Identification of Polyphenols by the Liquid Chromatography-Mass Spectrometry (LC-MS) Method

Identification of the polyphenol of extracts was carried out using an ACQUITY Ultra Performance LC^TM^system (UPLC^TM^) with binary solvent manager (Waters Corporation, Milford, MA, USA) and a Micromass Q-Tof Micro mass spectrometer (Waters, Manchester, UK) equipped with an electrospray ionization (ESI) source operating in negative mode. For instrument control, data acquisition and processing, MassLynx^TM^ software (Version 4.1) was used. Separations of individual polyphenols were carried out using a UPLC BEH C18 column (1.7 μm, 2.1 × 100 mm, Waters Corporation, Milford) at 30 °C. Samples (10 μL) were injected and elution completed in 15 min, with a sequence of linear gradients and isocratic flow rates of 0.45 mL·min^−1^. The mobile phase was composed of solvent A (4.5% formic acid, v/v) and solvent B (100% of acetonitrile). The program began with isocratic elution with 99% A (0–1 min), then a linear gradient was used until 12 min, lowering A to 0%; from 12.5–13.5 min, returning to the initial composition (99% A), and then holding constant to re-equilibrate the column. Analysis was carried out using full scan, data-dependent MS scanning from *m/z* 100–1500. The mass tolerance was 0.001 Dalton and the resolution was 5.000 Leucine enkephalin was used as the internal reference compound during ESI-MS accurate mass experiments and was permanently introduced via the LockSpray channel using a Hamilton pump. The Lock Mass Correction was +/−1.000 for Mass Window. All TOF-MS-chromatograms are displayed as Base Peak Intensity (BPI) chromatograms, and scaled to 12,400 counts per second (cps) (=100%). The effluent was led directly to an electrospray source with a source block temperature of 130 °C, desolvation temperature of 350 °C, capillary voltage of 2.5 kV and cone voltage of 30 V. Nitrogen was used as desolvation gas, with a flow rate of 300 L·h^−1^.

The characterisation of the individual components was carried out via retention time and accurate molecular masses. Each compound was optimized to its estimated molecular mass [M−H]^−^ in the negative mode before and after fragmentation. The data obtained from UPLC/MS were subsequently entered into the MassLynx 4.0 ChromaLynxTM Application Manager software. Based on these data, the software is able to scan different samples for the characterised substances.

The runs of polyphenolic compounds were monitored at the following wavelengths: ellagitanins at 240 nm, phenolic acid at 320 nm, flavonol glycosides and ellagic acids at 360 nm. Retention times (R_t_) and spectra were compared with those of pure standards. Calibration curves at concentrations ranging from 0.05–5 mg/mL (r^2^ ≤ 0.9998) were made for chlorogenic and neochlorogenic acid, *p*-coumaric acid, ellagic acid, quercetin-3-*O*-glucoside and -3-*O*-galactoside, luteolino-3-*O*-glucoside, apigenin-3-*O*-glucoside, and kaempferol-3-*O*-glucoside. The results was expressed as milligrams per g of dry matter (dm).

### 3.5. Analysis of Antioxidant Activity

The ABTS^°+^ activity of the sample was determined according to the method of Re *et al.* [27]. The total antioxidant potential of the sample was determined using a ferric reducing ability of plasma (FRAP) assay by Benzie *et al.* [28] as a measure of antioxidant power. A standard curve was prepared for all analyses, using different concentrations of Trolox. All determinations were performed in triplicate using a Shimadzu UV-2401 PC spectrophotometer (Kyoto, Japan). The results were corrected for dilution and expressed in milimoles of Trolox per gram dm (mmol TE/g dm).

### 3.6. Statistical Analysis

Results are given as the mean of at least three independent determinations. Hierarchal cluster (HA) was performed using STATISTICA v. 10 (Kraków, Poland) on mean values of three samples and 31 variables. An analysis of variance (ANOVA) and a multiple range test (Tukey’s HSD test) were carried out. Pearson’s correlations were determined using Microsoft Excel 2010. All analyses were performed in triplicates.

## 4. Conclusions

Inspection of these groups showed that the only individual of the subgroup represented by *R. grabowskii > R. pedemontanus >* *R. winmerianus and R. pomobustus* were reported as containing higher concentrations of phenolic compounds—mainly ellagitannins—and also antioxidant activity than the rest of the species belonging to this cluster. Generally, significant differences were found in phenolic compound content between the types of blackberry species.

Results also indicate that the wild blackberry leaves studied in this paper show great potential as an ingredient for the formulation of functional food products or for use in the cosmetic and pharmaceutical industries.
